# Consultations about randomised controlled trials are shorter and less in-depth for socioeconomically disadvantaged patients compared to socioeconomically advantaged patients: qualitative analysis across three trials

**DOI:** 10.1186/s13063-024-08216-4

**Published:** 2024-06-13

**Authors:** Mariana Popa, Bridget Young, Nikki Rousseau, Mary G. Cherry, Isobel Jenkins, Jane Cloke, Andrew Pettitt, Michael D. Jenkinson, Saiqa Ahmed, Allan R. Pemberton, Frances C. Sherratt

**Affiliations:** 1 Department of Public Health, Policy and Systems, University of Liverpool, Liverpool, UK; 2 School of Medicine, Faculty of Medicine and Health, University of Leeds, Leeds, UK; 3 Department of Primary Care and Mental Health, University of Liverpool, Liverpool, UK; 4 School of Medicine, Faculty of Health and Life Sciences, University of Liverpool, Liverpool, UK; 5 NIHR Applied Research Collaboration North West Coast, University of Liverpool, Liverpool, UK; 6 Department of Molecular and Clinical Cancer Medicine, University of Liverpool, Liverpool, UK; 7 The Clatterbridge Cancer Centre NHS Foundation Trust, Liverpool, UK; 8 Institute of Systems, Molecular and Integrative Biology, The University of Liverpool, Liverpool, UK; 9 The Walton Centre NHS Foundation Trust, Liverpool, UK

**Keywords:** Randomised, Inclusivity, Socioeconomic, Disadvantage, Communication, Equipoise, Trial, Qualitative

## Abstract

**Background:**

Patients from socioeconomically disadvantaged backgrounds are underserved in randomised controlled trials, yet they experience a much greater burden of disease compared with patients from socioeconomically advantaged areas. It is crucial to make trials more inclusive to ensure that treatments and interventions are safe and effective in real-world contexts. Improving how information about trials is verbally communicated is an unexplored strategy to make trials more inclusive. This study examined how trials are communicated verbally, comparing consultations involving patients from the most and least socioeconomically disadvantaged areas.

**Methods:**

Secondary qualitative analysis of 55 trial consultation transcripts from 41 patients, sampled from 3 qualitative studies embedded in their respective UK multi-site, cancer-related randomised controlled trials. Patients living in the most and least socioeconomically disadvantaged areas, defined using English Indices of Multiple Deprivation decile scores, were purposively sampled. Analysis was largely thematic and drew on the constant comparison method.

**Results:**

Recruiters communicated clinical uncertainty in a similar way for patients living in different socioeconomic areas. Consultations with disadvantaged patients were, on average, half the duration of those with advantaged patients, and tended to involve recruiters providing less in-depth explanations of trial concepts, used phrasing that softened trial arm risks, and described trial processes (e.g. randomisation) using informal or metaphorical phrasing. Disadvantaged and advantaged patients differed in the concerns they expressed; disadvantaged patients voiced fewer concerns and asked fewer questions but were also less likely to be invited to do so by recruiters.

**Conclusion:**

Interactions about trials unfolded in different ways between patients living in different socioeconomic areas, likely due to both patient- and recruiter-related factors. We present considerations for recruiters when discussing trials with patients from socioeconomically disadvantaged backgrounds, aimed at enhancing trial communication. Future research should examine disadvantaged patients’ and recruiters’ experiences of verbal trial communication to inform guidance that addresses the needs and preferences of underserved groups.

**Supplementary Information:**

The online version contains supplementary material available at 10.1186/s13063-024-08216-4.

## Background

Patients from socioeconomically disadvantaged backgrounds are one of several persistently underserved groups in clinical research [1]. Underserved patient groups are characterised by lower inclusion in research than one would expect from population estimates and a high healthcare burden that is not matched by the volume of research designed for patients from such groups [1]. Improving the inclusion of patients from socioeconomically disadvantaged backgrounds in clinical trials would increase the generalisability of trial findings to the intended broader population. It would also help to ensure that treatments and interventions tested in trials are effectively and appropriately delivered to the people who need them most and, therefore, reduce health inequalities and the burden on public services [2].

The term ‘socioeconomically disadvantaged’ is often used to refer to individuals who live in less favourable social and economic circumstances than the majority of others in the same society [3]. There is a lack of consensus as to how socioeconomic status should be operationalised, although it is commonly indicated by education, social class or income [4]. It is well established that those living in the most socioeconomically disadvantaged areas are more likely to experience poor health and reduced life expectancy, compared with those living in the least socioeconomically disadvantaged areas [5, 6]. To illustrate the extent of such inequalities, in 2018–2020, males living in the most disadvantaged areas of England lived 9.7 years fewer than males living in the least disadvantaged areas, with the difference at 7.9 years for females; furthermore, the gap in life expectancy at birth has risen for both sexes since 2015–2017 [7].

Globally, major research funders, such as National Institute for Health and Care Research (NIHR), United States National Institutes of Health and the Canadian Institutes of Health Research have acknowledged that researchers have not attracted a sufficiently diverse range of people to take part in clinical research and that further action is needed to improve equality, diversity and inclusion in research [2, 8, 9]. For example, NIHR initiated the ‘Innovations in Clinical Trial Design and Delivery for the Under-served’ (INCLUDE) project, which broadly aims to identify barriers and drivers to inclusion, as well as facilitate innovations in trial design and delivery [10].

Making research more inclusive to under-served groups and improving how trials are communicated to patients are major research priorities [11]. It has long been established that written trial communication is often inaccessible for many patients except those with high educational attainment [12], as it tends to include text at a higher literacy level than that of the average reader [13]. Although patient information sheets are a valued decision-making aid, most recipients place greater value on face-to-face discussions than they do on written information and would not consider participating in a trial without a personal approach [14]. By making changes to the content and presentation of verbal trial information, it is possible to improve patient understanding and willingness to be randomised [15–17]. This highlights the need to examine verbal trial communication with patients from different socioeconomic areas to inform how to optimise trial discussions, thereby enhancing informed consent and increasing recruitment of disadvantaged patients.

Qualitative studies embedded within trials have helped to improve trial recruitment and enhance informed consent for patients in general [15]. These studies usually analyse audio-recorded trial consultations, sometimes supplemented with patient interviews and/or trial recruiter interviews, to identify and address challenges that recruiters encounter in communicating trials through feedback. However, extending such an approach to improve trial communication and recruitment of patients from socioeconomically disadvantaged areas has not been investigated. As a first step to explore this, we examined if and how verbal trial communication varies by patient socioeconomic status. We conducted a secondary qualitative analysis of trial consultations from three qualitative studies embedded in their respective cancer-related randomised controlled trials.

## Methods

### Design

This was a secondary analysis of existing trial consultation repositories of qualitative data collected from three multi-site cancer-related randomised controlled trials. All three qualitative studies were embedded in their respective clinical trial. The primary aims of these qualitative studies were to identify trial recruitment barriers and inform strategies to enhance trial communication and/or design. Ethical approval to conduct the qualitative aspects of this research was obtained through each individual trial (PETREA 17/NW/0512; ROAM 15/NE/0013; LiTEFORM 17/WM/0096). The Standards for Reporting Qualitative Research (SRQR) [18] was used to guide the design and reporting of this study.

### Qualitative data sharing

The current study was led by University of Liverpool. Two of the trials had been led from Liverpool with members of the study team as trial investigators. To extend the sample, a data sharing request was made via the MRC-NIHR Trial Methodology Research Partnership working group. Trial 3 was accessed via this route. No other trials were identified which had recorded recruitment consultations and (a) had consent for data sharing and (b) had recorded information (i.e. postcode) that enabled direct or indirect identification of socioeconomic status (using English Index of Multiple Deprivation (IMD) deciles). Approval for participation of Trial 3 was obtained from the sponsor (Newcastle Hospitals NHS Foundation Trust) and a data sharing agreement was drawn up.

### Trials overview

The trials included in the analysis are summarised in Table 1. All were publicly funded cancer-related trials in UK secondary care settings, comparing markedly different management pathways, typically including monitoring or no further treatment as comparators. The qualitative studies were embedded in each of the trials from the start, continuing throughout the preliminary phase of trial recruitment. Sites for all three trials received training on the qualitative study at the start and during the trial, focused on optimising trial communication.

**Table 1:** Background to clinical trials in which qualitative studies were embedded

Trial identifier	Clinical speciality	Trials arms	Duration of consultation data collection	No. of NHS sites that contributed consultation data
Trial 1 (ISRCTN71502099) [19]	Oncology (brain)	Radiotherapy vs monitoring	31 months	13 (of 20 trials sites)
Trial 2 (ISRCTN86739591) [20]	Oncology (follicular lymphoma)	Guided by results of PET-CT: For patients with a complete metabolic response to induction: standard drug treatment vs monitoring For patients with a partial response to induction: standard drug treatment vs “test” drug plus standard drug treatment	30 months	30 (of 50 trial sites^a^)
Trial 3 (ISRCTN14224600) [21]	Oncology (head and neck)	Active laser therapy vs inactive laser therapy	18 months	6 (of 9 trial sites)

^a^At the time of writing as trial recruitment ongoing. See glossary for further explanation of terms

### Data collection

Further information regarding data collection methods for the three qualitative studies are detailed elsewhere [21–23]. In short, across the three trials, patients attended a clinic consultation at which recruiters would diagnose the patient or reiterate their diagnosis and/or discuss treatment results (Trials 1 and 2) or treatment plan (Trial 3) before discussing the trial. Immediately before discussing the trial, the recruiter requested the patient’s permission to audio-record the trial discussion and obtained written consent following the consultation. Patients who expressed an interest in discussing the trial further were often invited to attend further consultations, which were also audio-recorded where possible. Audio-recordings were transcribed verbatim, including non-verbal fillers, stutters, etc. but not pause or silence durations.

### Secondary data sampling

Across the three qualitative studies, 128 audio-recorded consultations with 106 patients were collected between 2017 and 2020. We purposively sampled a sub-set of patients living in the most and least disadvantaged areas from this pool of transcripts, using the English Index of Multiple Deprivation (IMD) [24]. The IMD uses seven domain indices and the Income Deprivation Affecting Children and Older People Indices to relatively rank deprivation levels in small areas of England (or lower-layer super output area (LSOA)) linked to an individual’s postcode [25]. Postcodes in England are ranked from most deprived (1) to least deprived (34,844). These ranks are also categorised into deciles 1–10, from most to least deprived.

We purposively sampled patients living in the most deprived deciles (1–3) and least deprived deciles (8–10). For brevity, we refer hereon to patients living in the most deprived LSOA area deciles as ‘disadvantaged’ and patients living in the least deprived LSOA area deciles as ‘advantaged’. We excluded consultations from patients who met any of the following criteria: (1) patient’s postcode produced no output data from IMD database (e.g. patient lives outside of England), (2) patient refused for their pseudonymised transcript to be used for further research purposes and (3) patient’s LSOA (based on postcode) is linked to IMD deciles 4–7.

We aimed to achieve a sample that was inclusive of patients from the most and least socioeconomically disadvantaged areas in England (using IMD deciles derived from postcodes), trials, recruiters and NHS sites within each of the trials to demonstrate the wider applicability of the findings. We were also keen to avoid sampling a disproportionate number of consultations from a particular recruiter who may be based at a hospital site that sees a disproportionate number of patients from certain socioeconomic backgrounds. We therefore used a sampling matrix to support us in linking a recruiter to at least one advantaged patient and one disadvantaged patient where possible. The use of the sampling matrix enabled us to carefully monitor the spread of patient socioeconomic status by trial and recruiter.

As a research team, we agreed to omit unusually short consultations (e.g. 3-min long) and those that entailed non-trial related discussions or recruiters largely reading from consent form points from the analysis because they offered minimal trial communication data to analyse. Sampling for consultations ceased when ‘data adequacy’ was reached (i.e. further consultation analysis no longer contributes to new findings) [26].

### Secondary data analysis

A female qualitative researcher (MP) with a psychology background led the analysis and developed the coding framework, with support from another female qualitative researcher (FS) with a health research background. Transcripts were imported into the qualitative analysis computer programme, QSR International NVivo [27] to assist the researchers with data indexing and coding.

Transcript analysis was largely thematic [28] and drew on elements of content analysis [29]. MP followed an iterative process of reading and re-reading transcripts to familiarise herself with the data, generating and refining a coding framework, searching for themes, reviewing themes and defining and naming themes. A hybrid approach of inductively and deductively coding transcripts was adopted to ensure rigour [30]. Some a priori codes were added to the coding framework before the transcripts were coded. These were informed by the literature on trial communication and clinical communication focused on social class, as well as input from the patient and public involvement (PPI) group (see below). MP then generated and refined further codes when coding the transcripts. Codes and themes were iteratively refined or combined to enhance one another [31].

To further explore the way themes were presented and their occurrences, MP drew on elements of content analysis [29]. MP used NVivo to explore patterns in key themes by patient socioeconomic status. This entailed defining the coding unit (i.e. themes), labelling transcripts by patient socioeconomic status and linking them to the specific trial and examining theme occurrences by patient socioeconomic status to explore differences and similarities. MP and FS held weekly project meetings to discuss patterns in the data, divergent cases and potential researcher preconceptions or biases, which enabled investigator triangulation [32, 33] and ensured analytical rigour [18, 34]. FS additionally used NVivo to examine theme occurrences by patient socioeconomic status to bolster reliability [29]. FS, NR, IJ and BY read a sub-set of transcripts and met on several occasions during the project to develop and refine the analysis.

In this paper, we often semi-quantify differences and similarities in themes that we identified (e.g. describing direction of trends) to provide greater clarity and improve the transparency of results, but we avoided listing specific frequencies because it could misrepresent the data, for example if a recruiter raises a topic inconsistently across consultations, and it could also detract from the detailed and nuanced data collected that we comment on [35].

### Patient and public involvement

A PPI group was assembled for the study. An advert was compiled describing the scope of the project and inviting ‘people from diverse backgrounds who are enthusiastic about widening participation in research… especially those who have previous experience of being treated for cancer’ to enquire about joining the study group. The advert was distributed to a patient contributor mailing list held by NIHR Applied Research Collaboration North West Coast. Respondents contacted FS by telephone or email. Respondents who provided relevant information about why they were suitable for the role were invited to join the PPI group, which ultimately included four people.

Members of the research team (MP and FS) facilitated two meetings with the PPI group. During the first meeting, themes identified from the literature were presented to the PPI group before coding the data to identify other potential areas to focus analytic enquiry. The study team met once more with the PPI group towards the end of the study to present the key findings, discuss dissemination routes and inform the development of a future funding application linked to the current study. Two public contributors (also co-authors) reviewed a draft of this paper and informed its development.

## Results

### Overall participant and consultation characteristics

The sample included 41 patients across the 3 trials from 20 UK sites. Table 2 summarises the key participant and consultation characteristics. Overall, 56% (*n* = 23) of the sample were living in the most advantaged areas and 44% were living in the most disadvantaged areas (*n* = 18).

**Table 2:** Consultation data characteristics

Participants	*N* = 41
Patient age, median (range)	60 (29–79) years^a^
Patient gender, males (females)	22 (19)
Patient Index of multiple deprivation decile^b^	
Disadvantaged (1–3)	18
Advantaged (8–10)	23
Trial consultations	*N* = 55
Trial 1 Qualitative participants	19
No. of consultations (initial vs secondary)	20 (18 vs 2)
No. of NHS sites	7
Trial participation status, consent (declined)	9 (10)
Initial consultation duration, median (range)	16 (3–49) min
Trial 2 Qualitative participants	16
No. of consultations (initial vs secondary)	29 (16 vs 13)
No. of NHS sites	9
Trial participation status, consent (declined)	15 (1)
Initial consultation duration, median (range)	16 (3–37) min
Trial 3 Qualitative participants	6
No. of consultations (initial vs secondary)	6 (6 vs 0)
No. of NHS sites	4
Trial participation status, consent (declined)	^a^
Initial consultation duration, median (range)	9 (5–24) min

*NHS* National Health Service

^a^Data missing for Trial 3 (see in-text detail)

^b^The Index of Multiple Deprivation ranks every small area in England from 1 (most deprived area) to 32,844 (least deprived area). The deciles are derived from ranks and we divided these into most deprived (1–3) and least deprived (8–10)

For the combined sub-samples linked to trials 1 and 2, the median participant age was 60 years (range 29–79) and most (69%) participated in their respective trial. Although data on age and trial participation status was not available for participants from Trial 3’s qualitative study, the mean patient age for participants on Trial 3 was 59.4 (8.8 SD). Disadvantaged patients had initial consultations that were on average half the duration of advantaged patients (median = 11 min (range 3–43), compared with 22 min (range 6–49)). There were slightly more males (*n* = 22, 54%) than females.

### Qualitative findings

Key qualitative findings are presented under two overarching headings: ‘presentation of key trial concepts’, which focuses on how recruiters communicated core trial concepts, such as uncertainty and equipoise, randomisation, and voluntariness and right-to-withdraw, and ‘gauging patient information needs’, which focuses on patterns in patient question-asking and recruiter efforts to check patient understanding.

Quotes shown are illustrative and representative of the findings, with associated identifiers (P = Patient’s consultation identifier, socioeconomic area = advantaged or disadvantaged, and trial number linked to Table 1 included for context).

### Presentation of key trial concepts

#### Conveying uncertainty and equipoise

Recruiters emphasised that there was uncertainty in the clinical community about the most appropriate treatment pathway and offered similar explanations about this to advantaged and disadvantaged patients:

Recruiter: *We genuinely don’t know at the moment—nobody knows the answer to whether people need this maintenance treatment.* (P29, Advantaged, Trial 2).

Recruiter: *It seems still an open question to see whether we could risk-adapt maintenance therapy, but we don’t know the answer.* (P40, Disadvantaged, Trial 2).

Irrespective of patient socioeconomic background, recruiters uniformly listed potential benefits of participating in a trial, including close follow-up, additional scans, access to treatments that were not available as standard care and improved health outcomes. They also highlighted similar drawbacks to participating in a trial, including undergoing further tests or procedures, trial arm risks, additional paperwork and more hospital visits.

Although recruiters listed similar potential benefits and drawbacks to participating in a trial to both advantaged patients and disadvantaged patients, there were key differences in how trial arm risks were presented.

Recruiters provided advantaged patients with detailed explanations of risk components, such as side effects. In particular, they specified individual treatment side effects and elaborated on the mechanisms for such side effects. In comparison, recruiters described risks to disadvantaged patients briefly and broadly:

Recruiter: *There can be additional side-effects to the combination of rituximab and lenalidomide, fundamentally more suppression of the immune system, so an increased risk of infection, low blood counts. There are other rarer side-effects of lenalidomide, including skin rashes, upset to liver function, upset to kidney function, and, most importantly, with lenalidomide, upset to the unborn child.* (P32, Advantaged, Trial 2).

Recruiter: *We also know that it does come at the risk of potential side effects… carrying on with the antibody drip for two years suppresses the immune system further, and some people will go on to get significant infections and other problems.* (P40, Disadvantaged, Trial 2).

Furthermore, whilst recruiters presented disadvantaged patients with information on treatment risks, they tended to soften how the risk was presented, for example, mentioning a risk but then explaining that it was unlikely or disassociating it with the potential cause:

Recruiter: *But as I say, the risk of getting bad infections and problems with this treatment actually is fairly small.* (P37, Disadvantaged, Trial 2).

Recruiter: *In the longer term the radiotherapy can affect your memory and your concentration a little bit, but that’s sort of over years. Um, difficult sometimes to know whether that would have been the normal ageing process anyway.* (P23, Disadvantaged, Trial 1).

In one of the trials, presentation of the treatment arms appeared to vary with patient socioeconomic status, with more positive presentation of active monitoring and negative presentation of radiotherapy with advantaged patients. As described above, recruiters emphasised uncertainty in similar ways with patients from both groups. However, recruiters had extended discussions about the risks of radiotherapy with advantaged patients in this trial and also tended to describe active monitoring to advantaged patients as ‘standard care’ or the preferred treatment pathway outside of the trial:

Recruiter: *At the moment, the general UK practice and also internationally a lot, is that if the tumour’s been macroscopically removed if it’s all visible bits have been taken out, often people will elect for a period of surveillance and then wait for the tumour to come back and then have radiotherapy.* (P11, Advantaged, Trial 1).

#### Randomisation

Irrespective of socioeconomic background, recruiters indicated to patients that they would be randomised if they took part. However, beyond this, the ways that recruiters described randomisation tended to vary by patient socioeconomic status. Recruiters used randomisation metaphors with disadvantaged patients, such as *‘flip of a coin*’ or ‘*pulled out of a hat*’:

Recruiter: *If you went into the trial it takes the decisions out of your hands and out of my hands. It’s like tossing a coin, there’s a 50/50 chance of either having radiotherapy or not having…* (P19, Disadvantaged, Trial 1).

Recruiters avoided such metaphors with advantaged patients. In discussions with advantaged patients, they also tended to refer to how or why patients were randomised, including reference to bias and the use of a computer to allocate patients:

Recruiter: *And then what we’ll do is we’ll compare the two groups of people, and it’s the computer that decides which way you go… It’s nothing to do with me or your surgeon … but the idea is that then that takes out any bias that I might have…* (P10, Advantaged, Trial 1).

#### Voluntariness and right-to-withdraw

The voluntariness of participating in a trial was discussed in both groups. However, in consultations with disadvantaged patients, consenting to the trial was likened to ‘*an option*’ or ‘*signing a bit of paper*’. In one example, a recruiter likened the process of obtaining informed consent to buying a used car:

Recruiter:*We never take consent today, because obviously it’s a bit like, you know, a bit like buying a used car.*

Patient:*Buying a car? (laughs)*

Recruiter: *Yeah (laughs). So, we give you a cooling off.* (P21, Disadvantaged, Trial 1)

Recruiters did not use these kinds of informal terms or metaphors to describe the process of obtaining informed consent with advantaged patients.

In around a third of all patients’ consultations, recruiters informed patients of the opportunity to withdraw from the trial at any time. Although this occurred fairly equally between the two groups, there was suggestion of some differences in how opportunity to withdraw was presented between them. When speaking with disadvantaged patients, recruiters tended to describe withdrawing using informal terms, included information on how to withdraw (i.e. to discuss with the trial team) and reassured patients that they would not be treated differently by the trial team. In contrast, recruiters tended to refer to the patient’s ‘rights’ when discussing withdrawing with advantaged patients:

Recruiter: *People who enter a study are allowed to withdraw themselves from the study at any time, for any reason, and they’re not even obliged to tell us what that reason is… All we would do at that time is revert you to standard treatment and continue… you can withdraw at any time without affecting your medical or legal right.* (P32, Advantaged, Trial 2).

Recruiter: *Even if you sign the bit of paper, you can change your mind and if you do so, then you just have to let (recruiter) or one of the people in (hospital department) know that actually you’ve changed your mind about taking part in the study and it won’t alter the rest of your treatment at all… It’s absolutely fine to change your mind…* (P6, Disadvantaged, Trial 3).

### Gauging patient information needs

#### Patterns in patients’ questions and concerns

Recruiters tended to invite advantaged patients to ask questions about the trial more so than disadvantaged patients. Furthermore, the way patients were invited to ask questions differed between the two groups. With advantaged patients, recruiters tended to pause periodically throughout the consultation after explaining a particular aspect of the trial to invite the patient to ask a question or they proposed a question that the patient might like to ask at that point:

Recruiter: *Is there anything I’ve missed out? Anything I should be saying? Any immediate questions?* (P34, Advantaged, Trial 2).

Recruiter: *Some people ask in terms of kind of, “What are the logistics of this process going forward?”* (P29, Advantaged, Trial 2).

Recruiters were less inclined to periodically invite disadvantaged patients to ask questions and tended to wait until the end of the main discussion to invite questions or do so immediately prior to completing the consent form:

Recruiter: *I’m going to go through [the trial information] and then if there’s anything that you want to ask, or your daughter wants to ask, then feel free and what we’ll do is we’ll ask you to sign a consent form if you’re happy to go ahead with the study at the end.* (P6, Disadvantaged, Trial 3).

Overall, advantaged patients asked more questions and raised more concerns, particularly unprompted ones, compared with disadvantaged patients.

#### Content of patients’ questions and concerns

Both groups asked questions and expressed concerns about trial arm processes and side effects, such as ‘*What’s the side effects going to be?*’ Advantaged patients also asked about current or emerging data about trial arms or treatment options outside of the trial to try to establish the optimal treatment pathway:

Patient: *Does the current data collected so far, does that suggest a better route using the tablet, or not?* (P32, Advantaged, Trial 2).

Patient: *So the, what would be the treatment plan if I wasn't to join the study?* (P41 Advantaged, Trial 1).

Patients often expressed specific concerns based on their individual circumstances, but such concerns differed markedly between the two groups. Personal concerns raised by advantaged patients included anxieties about medical tests/procedures and the potential implications of trial/treatment commitments on work, holidays or travel:

Patient: *I do long distance flights four times a year… because I’m just thinking of plane germs, infection, that kind of thing. (P34, Advantaged, Trial 2).*

Patient: *I already feel I'm cognitively being, affected and that's one of my key anxieties going back… given my line of work*. (P41, Advantaged, Trial 1).

In contrast, personal concerns raised by disadvantaged patients, largely focused on potentially being unable to commit to and attend trial-related appointments (typically due to transport limitations), lack of social support if the patient suffered side effects associated with trial treatments and financial difficulties:

Patient: *We don’t drive, so we come in by public transport. Could these appointments be arranged so they're not too early for us? Is that possible?* (P37, Disadvantaged, Trial 2).

Patient: *And the other thing is, erm, I don’t have anyone… if someone is with me [during treatment], a family, a sister or someone, er, if I had this, things they can help me with like food and everything.* (P19, Disadvantaged, Trial 1).

Patient: *I’m worried about finances […] But obviously you and I cannot discuss those because you’re a doctor and I’m penniless.* (P21, Disadvantaged, Trial 1).

#### Checking in about understanding

Recruiters tended to check in about understanding of the trial with advantaged patients more so than disadvantaged patients. Advantaged patients were invited to self-validate their understanding through closed questions, such as ‘*Does that make sense?*’ In contrast, recruiters tended to use open-ended questions to try to check understanding with disadvantaged patients:

Recruiter: *‘So if you can just tell me, in a couple of sentences, what you understood?’ (P35, Disadvantaged, Trial 2).*

## Discussion

This study identified similarities and differences in how randomised controlled trials are communicated between recruiters and patients living in socioeconomically disadvantaged areas and those living in socioeconomically advantaged areas. Recruiters were inclined to introduce clinical equipoise and the benefits of participating, similarly between the two groups. Overall, consultations with disadvantaged patients were on average, half the duration of those with advantaged patients. Recruiters tended to provide less in-depth explanations of trial concepts for disadvantaged patients, used phrasing that softened trial arm risks and described trial concepts or processes using informal or metaphorical phrasing. Compared with advantaged patients, disadvantaged patients asked fewer questions, expressed fewer concerns and tended to express concerns about barriers associated with social and economic factors.

Whilst equality is about equal distribution of resources so that each individual receives the same, equity is about fairness and justice [36] and entails distributing resources according to need to achieve equality [6]. In the current study, many of the observed patterns in communication may have been due to recruiters’ attempts to tailor communication and respond in equitable ways to meet individual patients’ communication needs. Tailoring clinical communication involves combining strategies and information intended to meet the needs and preferences of an individual patient based on their unique characteristics, related to the outcome of interest and derived from an individual assessment [37]. Tailoring to an individual patient’s needs improves patient satisfaction [38, 39], patient understanding [40] and health outcomes [41]. Although no empirical studies have examined verbal trial communication in the context of patient socioeconomic status, research has shown that patient socioeconomic status can influence patient–doctor interactions outside of the context of trials [42, 43]. For example, disadvantaged patients have been found to participate less actively in consultations and are less likely to volunteer information unprompted, whilst health professionals have been found to provide less information, spend less time building rapport, and listen less attentively with such patients [44]. This indicates that a patient’s socioeconomic background influences both how they communicate with health professionals and how health professionals communicate with them. The theory of cultural health capital [45] aims to illuminate how patient–practitioner interactions can play out in ways that generate disparities in health care and is influenced by both patients’ and practitioners’ cultural resources, assets and interactional styles [46]. Future research needs to examine trial communication experiences of both patients from underserved groups, and recruiters, to inform the development of trial communication guidance aimed at making research more inclusive. The theory of cultural health capital could also be usefully applied to inform future research in trial communication with underserved groups, as it provides a framework to explore and understand the ways in which cultural resources may be valued, leveraged and exchanged by patients and recruiters to influence how inclusive trials can be.

Our study suggests that trial recruiters may face previously unreported challenges conveying clinical equipoise with disadvantaged patients, as they provided disadvantaged patients with less in-depth information regarding trial arm risks and used phrasing that softened the risks (i.e. by providing additional reassurances that risks were low or unlikely) compared with advantaged patients. Previous research has shown that despite recruiters’ best intentions, they frequently lack balance in how they present trials arms during consultations, which can hamper efforts to convey equipoise effectively for all patient groups [47]. As disadvantaged patients are at higher risk of health anxiety [48], it is possible that recruiters are tailoring risk communication to avoid triggering anxiety or attempting to alleviate fear amongst patients whom they judge to be at risk of experiencing increased health anxiety [49]. There is also an indication from one of the trials that recruiters might make conscious or unconscious judgements about individual patients, linked to socioeconomic characteristics, that lead to communication being loaded towards a specific trial arm. As this finding was evident in only one of the trials and various factors may have influenced how trial arms were presented to patients, further research is needed to explore this possible association.

Spending more one-to-one time discussing trials with patients has been found to be one of the most effective strategies to improve patient understanding [50, 51]. Since socioeconomically disadvantaged patients exhibit lower levels of understanding and health literacy than advantaged patients [52, 53], we anticipated that trial consultations with disadvantaged patients would have been longer and more in-depth than those with advantaged patients; this was not the case in the current study. The framework of cultural health capital [45] may also help to explain why trial consultation durations in the current study were shorter with disadvantaged patients. Outside of the context of trials, disadvantaged patients have been found to seek out less treatment-related information compared with advantaged patients [54], whilst it has been suggested that health professionals often assume that disadvantaged patients prefer less detail during health consultations compared with advantaged patients [42, 43]; thus, demonstrating how the reciprocal influence of patients and recruiters result in different consultation patterns in the current study. This also aligns with patterns we observed in patient question-asking, with disadvantaged patients tending to ask fewer questions and health professionals tending to provide disadvantaged patients with fewer opportunities to ask questions.

In the present study, recruiters used informal language with disadvantaged patients to describe trial processes. For example, recruiters tended to use descriptions of randomisation that included (often gambling-related) metaphors with disadvantaged patients (e.g. ‘*flip a coin*’). Previous research suggests that tailoring communication to an individual’s health literacy has been shown to improve patient understanding, whereas using simplistic language with all patients did not improve patient understanding [55]. The current findings support the proposal that trial consultation interactions are facilitated by the health professional’s knowledge or assumptions about the patient’s social context and health literacy [56]. However, taking the above communication about randomisation as an example, patients have been found to prefer descriptions of randomisation that avoid gambling-related metaphors [57, 58]. Such metaphors can lead patients to perceive that they might ‘win’ or ‘lose’ in the randomisation process and have been found to impede trial recruitment [59]. A key limitation of the current literature on trial communication is that largely, researchers have not collected and/or reported patient socioeconomic characteristics, and when such characteristics are reported, underserved groups are not adequately represented in study samples. This highlights the need for future work to explore trial communication needs and preferences across various underserved groups to inform future communication guidance.

Informed by the current study results, we have summarised considerations to enhance how trials are communicated with patients from socioeconomically disadvantaged groups, in order to make trials more accessible to such patients (Table 3). These considerations may be particularly useful for recruiters working on trials involving populations that are likely to consist of a large proportion of patients who are socioeconomically disadvantaged. We have also summarised opportunities for trial methodologists to improve trial communication for disadvantaged patients, including priority areas for future research in this area (Table 4).

**Table 3:** Considerations to make clinical research more accessible to patients from socioeconomically disadvantaged areas by optimising verbal trial communication

(1) Previous evidence suggests that extending discussions with patients can enhance their understanding of trials [50, 51], yet the current study found that discussions with disadvantaged patients were shorter and in less-depth than those with advantaged patients. It is important to provide disadvantaged patients with adequate time and space to discuss the trial and avoid consultations being prematurely curtailed
(2) In the current study, disadvantaged patients asked fewer questions and were given fewer opportunities to ask questions. Periodically providing disadvantaged patients with support and opportunities to ask questions throughout a consultation and checking understanding will enhance their understanding
(3) Provide disadvantaged patients with adequate information about trial arm risks to improve informed consent, whilst offering additional explanation or reassurance as appropriate to avoid them from becoming unduly concerned about risks
(4) Avoid language that might be seen to trivialise trial participation or processes, such as using gambling metaphors to describe randomisation [57, 58]
(5) Patients from socioeconomically disadvantaged backgrounds may raise additional personal concerns (e.g. financial, social, etc.) during trial consultations. Showing interest in these concerns and discussing strategies to overcome barriers (where possible) will help disadvantaged patients to feel their concerns are taken seriously and support them in deciding whether to participate in the trial

**Table 4:** Opportunities for trials methodologists to improve trial communication for patients from socioeconomically disadvantaged backgrounds

(1) Examine the reporting of data on patient socioeconomic status and other intersecting patient characteristics in the trial communication literature to consider whether findings may or may not be generalisable to a broad population
(2) Conduct future research with patients from socioeconomically disadvantaged backgrounds to (a) establish patients’ trial communication preferences and needs, and (b) explore the extent to which different recruiter trial communication strategies enhance understanding (or otherwise) amongst patients from socioeconomically disadvantaged backgrounds
(3) Conduct future research with recruiters working on clinical trials that involve socioeconomically disadvantaged populations to understand recruiters’ trial communication goals and intentions in relation to the observed differences in the current study, as well as the challenges to communicating equitably
(4) Ensure patients from socioeconomically disadvantaged backgrounds are included in patient and public involvement in research on trial communication
(5) Develop and implement guidance to support recruiters to optimise trial communication to make research more accessible to patients from socioeconomically disadvantaged backgrounds
(6) Conduct further research to examine whether recruiters make judgements about individual patients, linked to patient socioeconomic characteristics, that lead to communication being loaded towards a specific trial arm

### Strengths and weaknesses

This is the first study to examine similarities and differences in verbal trial communication between patients living in the most socioeconomically disadvantaged areas and patients living in the least socioeconomically disadvantaged areas. A key strength of this study is that we analysed 55 consultations from 41 patients living in the most and least socioeconomically disadvantaged backgrounds (based on IMD) across three cancer-related trials. This study’s sample size was guided by the principles of ‘information power’ [60] and is typical for a qualitative study designed for in-depth consultation analysis that is crucial to generate insights concerned with patterns in trial communication [15]. IMD is a widely used area-based measure of socioeconomic status; however, future primary research of this nature should look to supplement IMD with an individual-based measure (e.g. education level) to provide a more rounded reflection of individual participants’ socioeconomic background. It was not possible to do so in the current study due to this being a secondary analysis. Qualitative studies embedded within trials typically entail analysing audio-recorded trial consultations to inform strategies to enhance communication and recruitment [15]. A persistent challenge in analysing consultation data is the number of variables that can influence communication (e.g. recruiter communication style, recruiter role, whether significant others are present etc.) We developed and followed a sampling matrix to achieve a sample that included a range of recruiters and trials matched by patient socioeconomic status, with the aim of establishing patterns that are likely to be more widely applicable. This study involved qualitative data sharing, which was facilitated via the MRC-NIHR Trial Methodology Research Partnership (TMRP). Although there are some examples of analysing qualitative datasets cross-trial, these have typically been conducted within connected research groups (i.e. studies based within one research group or studies between groups that share an investigator) (e.g. [47, 61]). There are currently very few examples of cross-trial qualitative data analysis undertaken by unconnected research teams likely due to the additional barriers that data sharing of this nature presents [62]. Although several researchers within the MRC-NIHR TMRP expressed an interest in collaborating, and they had patient consent to share data, opportunities to collaborate were limited as most researchers had not collected/retained data on certain patient socio-demographics, such as socioeconomic status. Including Trial 3 in the present study increased the pool of consultation transcripts, the variety of trials examined and the organising institutions. However, qualitative data sharing presented some challenges. To ensure adequate anonymisation, the investigator for Trial 3 (NR) re-checked all study transcripts line-by-line prior to sharing, which was time-consuming. Several members of the Trial 3 team were no longer working at the same institute by the end of this analysis, which resulted in difficulties accessing additional data to aid the analysis—hence missing data for some variables (see Table 2). Although we had fewer consultations to draw on from Trial 3 due to varied site engagement and trial recruitment difficulties [63], across the three trials, we obtained a diverse sample overall in terms of patient socioeconomic status, age, gender, randomisation, trial allocation arms and NHS sites. However, we did not have access to data regarding some other intersecting variables, such as patient ethnicity, as they were not collected in the primary research studies. Consent rates differed substantially by trial, which is reflected in Table 2. Although a patient’s interest in the trial would influence the nature of the consultation and duration, this should not influence our key findings as patients were sampled from both advantaged and disadvantaged groups across all three trials.

As a research team, we have reflected on how our own socioeconomic backgrounds and beliefs could prompt preconceptions or biases when analysing the data. In order to minimise bias, MP kept a reflexive journal and MP and FS had regular analysis meetings, providing opportunity to reflect and act upon any preconceptions or biases that arose during coding and writing [64].

Our results provide insights into patterns of communication between recruiters and patients living in different socioeconomic backgrounds. We do not know how patients felt about their recruitment consultations. Future research should include patient interviews, alongside audio-recorded recruitment consultations to understand how patients experienced the communication. Further work could inform the development of guidance to support recruiters in communicating effectively with patients from underserved groups. Finally, this secondary analysis focused on the content of what was said and the associated generated themes. Conversation analysis of full verbatim transcripts that also include details (such as attempts to ask questions, duration of pauses, etc.) could offer more insight about the nuances of communication in this context.

## Conclusions

This secondary qualitative analysis compared patterns in verbal trial communication between recruiters and patients living in different socioeconomic areas. The findings suggest that these complex interactions unfold in different ways between patients living in different socioeconomic areas, likely due to both patients’ and recruiters’ cultural skills, verbal and non-verbal competencies, attitudes and behaviours and interactional styles. We presented considerations to support recruiters to enhance verbal trial communication with patients from socioeconomically disadvantaged backgrounds, but the findings can also be used to guide the direction of future work that will inform the development of comprehensive guidance to improve how trials are communicated to underserved groups. Examining socioeconomically disadvantaged patients’ views and experiences of verbal trial communication, including those belonging to multiple underserved groups, is a key priority in developing such training or guidance. Further work should also aim to understand recruiters’ communication goals and intentions when communicating trials with patients from socioeconomically disadvantaged backgrounds. Improving verbal trial communication for underserved groups will empower patients to make informed decisions about their treatment and care, improve the inclusivity of trial recruitment and, therefore, ensure that trial treatments and interventions are safe and effective in real-world contexts.

### Supplementary Information

Supplementary Material 1.

## Data Availability

The datasets analysed during the current study are not publicly available due to the study being a secondary data analysis and the lead researcher not being the data custodian of the datasets used. However, the primary datasets may be available upon request from the respective primary study leads (Bridget Young and Nikki Rousseau). Further details are available by contacting the corresponding author.

## Glossary and abbreviations

Clinical equipoise: uncertainty about the relative clinical merits of the intervention arms in a trial [65].
Deciles: dividing a population up into ten equal groups, according to how certain values are distributed.
Oncology: study of cancer.
Monitoring: closely watching a patient’s condition but not actively treating them unless there is a change or worsening of their condition.
Radiotherapy: treatment that uses beams of intense energy usually to kill cancer cells. The beams are precisely aimed at the area that needs treating using a large machine.
PET-CT: positron emission tomography (PET) image combined with computerised tomography (CT). In the context of Trial 2, patients had a PET-CT scan to assess how effective their induction (first) treatment was and how the patient responded.
Complete metabolic response: complete metabolic response to induction treatment. A complete metabolic response indicates that induction treatment was highly effective, with no or minimal evidence of disease.
Partial response: partial response to induction treatment. A partial response indicates that induction was effective, but shows remaining residual disease that requires treatment.
Laser therapy: use of low power laser light therapy. In Trial 3, the treatment aimed to prevent a condition called ‘mucositis’ (pain and inflammation of the mouth) in patients who are undergoing radiotherapy for head and neck cancer.
PPI: patient and public involvement
NIHR: National Institute for Health and Care Research
INCLUDE: Innovations in Clinical Trial Design and Delivery for the Under-served
MRC-NIHR TMRP: Medical Research Council—National Institute for Health and Care Research Trials Methodology Research Partnership
PETREA: phase 3 evaluation of PET-guided, response-adapted therapy in patients with previously untreated, high tumour burden follicular lymphoma
LITEFORM: Lite Therapy Effectiveness For Oral Mucositis Trial
ROAM: radiation versus observation following surgical resection of atypical meningioma
IMD: indices of multiple deprivation
LSOA: lower-layer super output area
NHS: National Health Service
